# Establishing Core Outcome Sets to Advance Evidence Synthesis in Behavior Analysis

**DOI:** 10.1007/s40614-026-00507-2

**Published:** 2026-07-07

**Authors:** Alan Tennyson, Richard May, Aoife Mc Tiernan

**Affiliations:** 1 School of Psychology, University of Galway, Galway, Ireland; 2 School of Psychology, University of South Wales, Pontypridd, UK

**Keywords:** Core outcome sets, Evidence synthesis, Single-case experimental design, Behavior analysis, Outcome heterogeneity, Social validity

## Abstract

Applied behavior analysis is grounded in data-based decision-making and evidence-based practice (EBP). EBP is increasingly reliant on evidence syntheses (i.e., the combining of findings from multiple research studies to inform on a particular topic). Within this context, outcome heterogeneity is defined as variability in the outcome measures employed across research studies evaluating a particular intervention, including what is measured, how, and when. Outcome heterogeneity has been identified as a substantive barrier to evidence synthesis across health and social care, with direct consequences for clinical guideline development and policy. This article draws on examples from behavior-analytic and adjacent research to argue that outcome heterogeneity represents a methodological challenge for behavior analysis. A Core Outcome Set (COS) is a consensus-based, standardized set of outcomes that should be measured and reported as a minimum when evaluating a particular intervention. COS have been developed across a range of health behavior change interventions (e.g., for sedentary behavior, behavioral weight management, self-management of diabetes) as a structured response to the challenge of outcome heterogeneity. This article proposes increased engagement with the broader movement toward COS and argues that behavior analysis offers methodological resources not currently included in COS frameworks. Engagement with COS infrastructure should be approached with critical consideration, incorporating reflection on both its potential risks and benefits, as well as its alignment with the principles and practices of the field of behavior analysis. Risks of engagement, including the potential marginalization of idiographic measurement and inductive experiment techniques, are identified and mitigations proposed.

## The Problem of Outcome Heterogeneity

Evidence-based practice (EBP) is central to improving the quality of health and social care (Djulbegovic & Guyatt, 2017). Applied behavior analysis (ABA) contributes to this agenda through empirically supported interventions that reduce challenging behavior^1^ and promote meaningful skill development (Abraha et al., 2017; Eckes et al., 2023; Heyvaert et al., 2015; Neil et al., 2021; Novak & Honan, 2019). Its emphasis on data-based decision making and socially valid outcomes places ABA in close alignment with the contemporary EBP infrastructure in health research and policy (Slocum et al., 2014). Systematic reviews and meta-analyses, collectively referred to as evidence synthesis, provide the structured methods through which findings are integrated across studies and translated into evidence-based clinical guidelines, policy and service commissioning decisions (de Vet et al., 2011; Higgins et al., 2019; Sharp et al., 2022).

The utility of evidence synthesis depends on the comparability of outcomes reported across primary studies (Sprenger et al., 2025). When studies evaluating the same intervention measure different constructs, employ different instruments to assess those constructs, or assess at different time points, meaningful aggregation becomes difficult and conclusions are less transferable into practice (Higgins et al., 2019). This problem, referred to as outcome heterogeneity, has been reported as a substantive barrier to evidence synthesis across health and social care disciplines (de Vet et al., 2011; Williamson et al., 2017). Outcome heterogeneity can limit conclusions drawn from a body of evidence and therefore weaken the evidential foundations of clinical guidelines, thus reducing the influence of a research base on policy.

Given the importance of systematic reviews and meta-analyses, health research has developed an increasingly rigorous infrastructure for evidence synthesis, including structured review procedures, risk of bias tools such as RoB2 (Sterne et al., 2019), and the GRADE framework (Grading of Recommendations Assessment, Development and Evaluation) for rating the certainty of evidence and the strength of clinical recommendations (Guyatt et al., 2008). In parallel, initiatives promoting greater standardization of outcome domain selection and reporting, including the Core Outcome Measures in Effectiveness Trials (COMET) initiative and the International Consortium on Health Outcome Measurement (ICHOM), have gained considerable traction across health research. Systematic reviews and meta-analyses now serve as the primary evidentiary foundation for clinical guideline and policy development (Sharp et al., 2022). The standards embedded in this infrastructure, including how evidence is appraised for quality, how outcomes are selected for inclusion in reviews, and how findings are rated for certainty, increasingly determine which research contributes to guidelines and which is excluded or downgraded, independent of the quality of the individual studies.

As clinical guidelines and commissioning decisions become more tightly coupled with the outputs of evidence synthesis, engagement with outcome standardization should be a priority for the field of behavior analysis. Nonengagement risks marginalization in policy and clinical guideline development, not because behavior-analytic studies lack rigor, but because (1) outcome heterogeneity exists within the behavior-analytic literature and (2) outcome domains and measurement appraisal frameworks may be structured in ways that do not accommodate direct, idiographic measures or SCED conventions. This article examines the problem of outcome heterogeneity in behavior-analytic intervention research, drawing on early intensive behavioral intervention (EIBI) and positive behavior support (PBS) as primary case examples. It then considers how adjacent fields have addressed this problem through Core Outcome Sets (COS) frameworks, and whether those frameworks are applicable to behavior analysis. The article further argues that behavior analysis offers methodological resources to COS development, particularly in direct measurement, operational definition and single-case experimental (SCED) design conventions, that existing frameworks lack. Finally, the article identifies risks of engagement alongside proposed mitigations.

## Outcome Heterogeneity in Behavior-Analytic and Adjacent Fields

Evidence of outcome heterogeneity in behavior-analytic intervention research can be found in systematic reviews evaluating the efficacy of EIBI. A Cochrane review by Reichow et al. (2018) documented substantial variability across included studies in the outcome domains assessed, the instruments used to assess those domains, and the time points at which participants were evaluated. Outcome domains span adaptive functioning, symptom severity, communication skills, and parental stress, each measured using different tools and at varying follow-up intervals. The review team downgraded the quality of evidence for most outcomes due to this unexplained heterogeneity and concluded with a direct recommendation that future research should use consistent outcome measures (Reichow et al., 2018). The downgrading was not a judgement on the quality of the primary studies themselves. It reflected the cumulative effect of inconsistent outcome selection and reporting across an intervention literature that is otherwise among the most developed in behavior-analytic research.

The consequences of heterogeneity are further illustrated by Eldevik et al. (2009), whose meta-analysis of EIBI outcomes encountered substantial variability in the assessment instruments used across studies (Eldevik et al., 2009). Outcome scores were reported as standard scores in some studies, and age equivalents or raw scores in others. These inconsistencies complicate statistical synthesis and increase the risk of generating misleading or inflated effect estimates (Nugent, 2021). To manage this, Eldevik et al. restricted their analysis to studies reporting standard full-scale IQ scores only and standardized the follow-up period to two years. Although these restrictions were reported as methodologically necessary, they also substantially narrowed the evidence base available for synthesis. Studies that used alternative reporting approaches were excluded from the pooled analysis because measurement inconsistencies rendered their data incompatible.

The heterogeneity documented by Reichow et al. (2018) and Eldevik et al. (2009) is not confined to which outcome domains are selected. Inconsistency can also be identified within individual domains. Intelligence quotient is assessed using tests ranging from Stanford-Binet (Roid, 2003) to various editions of the Wechsler scales (Wechsler, 2012), each with differing age norms and scoring procedures, raising the question if the IQ reported in one study is the same as the IQ reported in another. Adaptive functioning commonly assessed using the Vineland Adaptive Behavior Scales (Sparrow et al., 2016), varies further depending on the edition used, who serves as the informant and whether composite or domain specific scores are reported. Language outcomes show similar diversity, with some studies using standardized instruments such as the Mullen Scales of Early Learning (Dawson et al., 2010) or the Reynell Development Language Scales (Edwards et al., 1999), and others relying on observational coding systems or interaction-based metrics (Ingersoll & Schreibman, 2006). Autism symptom severity is measured using tools as varied as the Autism Diagnostic Observation Schedule (Hus & Lord, 2014), the Childhood Autism Rating Scale (Chlebowski et al., 2010), often with study specific adaptations. Researchers, practitioners, and policy makers attempting to evaluate intervention effectiveness across this literature must therefore navigate non-equivalent and sometimes nonoverlapping metrics. Existing evidence synthesis processes are poorly equipped to resolve this heterogeneity without greater consistency in what is measured and how.

A similar pattern can be found in the PBS literature. A useful distinction here is between domain-level heterogeneity, where studies do not share the same underlying construct (e.g. Quality of Life, challenging behavior, or community participation) and instrument-level heterogeneity, where studies address the same construct using different measurement tools. The latter is not necessarily problematic for synthesis; where studies converge on the same construct, cross-instrument comparison can support generalizability rather than undermine it (Williamson et al., 2017). Within the PBS literature, some degree of construct-level convergence is evident: challenging behavior, for example, is a shared focus across a substantial proportion of studies, with instruments including the Aberrant Behavior Checklist (Aman et al., 1985), the Behavior Problems Inventory (Rojahn et al., 2001), and direct measures all focusing on the shared construct. Where studies share a construct of this kind, their findings are, in principle, amenable to synthesis even when measurement tools differ. The more acute synthesis problem in PBS arises from domain-level heterogeneity: studies assess challenging behavior (Durand et al., 2013; Kirkpatrick et al., 2019), adaptive functioning (Clarke et al., 2019; Ennis et al., 2020), and caregiver reports on quality-of-life indicators (Finn, 2020; Musetti et al., 2021), in varying combinations and without an agreed minimum set of domains. Variability is further compounded by inconsistent operational definitions applied to target behaviors, inconsistent timing of dependent measures and insufficient reporting of measurement procedures across studies. Heterogeneity is primarily visible in the choice of standardized assessment instruments within shared domains. PBS research shows heterogeneity at both levels, in what is selected for measurement and how it is measured and reported.

Beqiraj et al. (2022) provides a further account of the impact of outcome heterogeneity on evidence synthesis in PBS research, involving children and young people (Beqiraj et al., 2022). Their systematic review of 30 PBS intervention studies conducted in special education settings found outcome heterogeneity sufficiently extensive to preclude meta-analysis. Although some studies employed direct observation of a target response, the metrics reported (i.e., frequency, rate, latency and percentage reduction) varied considerably across studies. The authors reported that direct observational data were frequently paired with indirect ratings scales, unvalidated behavioral checklists, or operationally imprecise definitions of target responses. Observer training procedures, operationalization of target constructs, and interobserver agreement reporting were inconsistent across studies. The Beqiraj et al. review suggests that measurement practices in PBS have developed without sufficient coordination, creating difficulties in conducting evidence synthesis.

The consequences of absent outcome standardization are particularly evident in the randomized controlled trials (RCTs) evidence based evaluating PBS interventions for adults with intellectual disabilities experiencing challenging behavior. The most substantive trials conducted to date are: Hassiotis et al. (2018), which evaluated a manualized PBS approach, and McGill et al. (2018), which examined an intensive PBS support service model; together, these represent the largest and most methodologically developed RCT evidence available to inform policy and practice for this population. Across both trials, only one outcome measure is shared, the Aberrant Behavior Checklist (Aman et al., 1985), an indirect, proxy-rated measure that relies on caregiver or support staff ratings rather than direct observation of the target response. The absence of shared directly observed outcomes across these two trials is not a minor reporting inconsistency. It reflects a broader pattern in which the studies best positioned to influence clinical guidelines and commissioning decisions have been conducted without an agreed minimum standard of what should be measured.

Outcome heterogeneity is not unique to behavior analysis. Across the broader autism intervention literature, heterogeneity in outcome measurement has been extensively documented, with reported outcomes ranging from adaptive functioning and symptom severity to broader quality-of-life indices, assessed using various tools and at inconsistent time points (Lai et al., 2020; Rodgers et al., 2021; Speyer et al., 2022). Adjacent fields show similar difficulties with evidence synthesis as a result. For example, a Cochrane review by Williams et al. (2013) examining the efficacy of selective serotonin reuptake inhibitors (SSRI) for autistic people identified eighteen different outcome measures across the nine included trials, with minimal overlap in the domains assessed or instruments employed (Williams et al., 2013). Of the eighteen measures only one, the Clinical Global Impression Improvement Scale (Guy, 1976), was suitable for meta-analysis. The authors concluded that such inconsistencies constrained the reliability of any conclusions that could be drawn regarding the use of SSRIs.

As noted earlier, the practical consequences of outcome heterogeneity extend beyond the primary research and into the policy infrastructure that in turn shapes service provision. An example of this can be seen regarding the evaluation of evidence for pharmacological intervention for challenging behavior. In 2015, the UK’s National Collaborating Center for Mental Health applied the GRADE framework to rate the certainty of evidence across included interventions for challenging behavior (National Collaborating Center for Mental Health, 2015). Unexplained outcome heterogeneity was treated as inconsistency and was reported as a recurring reason for downgrading evidence to a low or very low quality. In the pharmacological evidence review, meta-analysis of naltrexone for self-injurious behavior was not possible because of heterogeneity of study design and outcome measures. This finding illustrates how outcome heterogeneity can render an entire body of knowledge unusable in the development of clinical guidelines for practice. Perhaps more concerning was that where empirical data were absent or could not be pooled, the guideline development group relied on a structured consensus process to generate recommendations. A field committed to data-based decision making should find it uncomfortable that the guidelines most likely to shape commissioning decisions for supporting those experiencing challenging behavior were, in part, based on expert consensus rather than evidence and that outcome heterogeneity was a contributing factor.

Not all heterogeneity is problematic for synthesis. When studies address the same construct using different instruments, cross-study instrument convergence can strengthen generalizability rather than undermine it, and a separate analysis for each distinct construct is methodologically appropriate rather than a symptom of inconsistency. As identified in the PBS research above, the synthesis problem becomes acute under more specific conditions: when studies do not share core outcome domains; when the same construct is operationalized using noncomparable metrics or assessment-time windows; or when reporting is insufficient to support comparability. Outcome heterogeneity is, moreover, one among several sources of variability in the SCED literature; differences in participant characteristics, implementation context, and the response-guided indicative nature of many single-case studies present additional challenges in evidence synthesis that outcome standardization alone cannot resolve. The goal of standardizing outcome domains and reporting conventions is not to suppress heterogeneity but to make it interpretable: a minimum set of shared domains supports principled explanation of between-study variation, allowing reviewers to ask why studies differ, rather than whether they can be brought together in evidence synthesis.

## Core Outcome Sets

Core Outcome Sets (COS) are a minimum, consensus-based set of outcome domains recommended for inclusion in all studies evaluating a given intervention, condition, or construct (Williamson et al., 2017). COS are intended to function as a reporting floor, rather than a ceiling. Idiographic outcomes remain available to researchers, because the COS specifies only that a minimum set of shared domains is reported alongside them. Developed initially within medical research, COS initiatives have since been established across a wide range of applied areas, including sedentary behavior (Curran et al., 2022), behavioral weight management (Mackenzie et al., 2020), and the self-management of diabetes (Nielsen et al., 2018).

The development of a COS typically begins with a preparatory systematic review to identify the outcome domains and measurement approaches used in the existing research, creating an initial long list of candidate domains (Williamson et al., 2017). This long list is then prioritized through structured consensus methods. Delphi surveys are commonly used for this purpose. In Delphi surveys, participants (drawn from stakeholder groups) complete successive rounds of rating, receiving summarized feedback after each round that allows them to reconsider their ratings in the light of the group’s responses. Quasi-anonymity is typically maintained throughout, where participants’ identities may be known to the research team, but individual judgments remain anonymous to other participants, reducing the influence of dominant voices and face-to-face social pressures (Hasson et al., 2000). Delphi surveys are followed by consensus meetings to confirm inclusion decisions and refine outcome definitions. Stakeholders, including people with lived experience of the intervention, family members, clinicians, researchers, and policymakers, are engaged throughout, ensuring that the resulting outcome set reflects the priorities of those most directly affected (Williamson et al., 2017). COS development projects are typically registered early with resources such as the COMET initiative database to improve transparency and reduce duplication. Finally, feasibility pilot studies are often conducted to assess the administrative burden of proposed domains before dissemination.

The Outcome Measures in Rheumatology (OMERACT) initiative is one of the earliest examples of COS adoption in health research. Prior to OMERACT, rheumatology trials reported a wide range of outcomes with little consistency across studies. Following the adoption of shared core domains, including pain and physical function, reporting became more consistent and cross-trial comparison more tractable (Boers et al., 2014). Greater consistency in reporting supported clearer meta-analyses and strengthened the evidence base for clinical recommendations (Kirkham et al., 2013). A more recent example from behavioral medicine is the STAR-LITE initiative (Mackenzie et al., 2020), which developed a COS for behavioral weight management interventions incorporating both physiological outcomes such as weight loss and body composition, and psychosocial outcomes, including quality of life. That a COS developed for behavioral medicine could accommodate outcomes of such different types, some which are more readily quantifiable and others more difficult to operationalize, is relevant to behavior analysis, where a comparable range of outcome types is encountered in intervention research.

As discussed earlier, outcome heterogeneity affects the literature evaluating autism interventions and supports. Joseph et al. (2024) developed a COS for autistic people using the International Consortium for Health Outcome Measurement (ICHOM) framework, formally titled the Autism Spectrum Disorder COS in line with the diagnostic nomenclature adopted by the working group (Joseph et al., 2024). ICHOM is an international nonprofit organization that develops standardized outcome sets for specific health conditions, with the aim of enabling comparison of outcomes across health-care systems, research contexts, and countries. Unlike COMET, which functions primarily as a registry and methodological resource for COS development, ICHOM produces its own condition-specific outcome sets through structured working groups and positions these sets as standards for adoption across research and clinical practice internationally. The ICHOM autism COS was developed by a working group spanning 13 countries, including behavior analysts, researchers, clinicians, policy makers, and people with lived experience. Using a modified Delphi-based consensus process across surveys and structured discussions, the working group identified a minimum set of core outcome domains: core symptoms (social communication, restrictive and repetitive behaviors), daily functioning (adaptive skills, leisure), accessibility and supports (quality of life, family functioning), and additional domains including emotional regulation, anxiety, and sleep. The set also specifies key variables such as age and co-occurring conditions, intended to support risk adjustment and improve interpretability across groups of people who differ in age, diagnostic profile, and support context, thereby allowing outcomes to be compared in a way that accounts for participant-level variation rather than assuming heterogeneity across the intervention literature. This will ultimately improve evidence synthesis for autism supports and interventions that will support the development of evidence informed policy and practice. Given the extent of the literature on behavior-analytic supports for autistic people, this will be highly beneficial to the field of behavior analysis; however, there is scope for a more comprehensive adoption of COSs in the field across varying applications.

## Core Outcome Sets and Behavior Analysis

A principle of COS development is the involvement of key stakeholders throughout the outcome selection process (Williamson et al., 2017). In medical and allied health research, this is formalized as Patient and Public Involvement (PPI), and it is increasingly expected by UK funders of health research (Bee et al., 2018). A review of COS initiatives found that 87% of the studies reviewed reported inclusion of people with direct lived experience or their representatives (Biggane et al., 2018). Wolf (1978) described social validity across three domains: the social significance of intervention goals, the acceptability of intervention procedures, and the importance of outcomes to those receiving the intervention, a framework that echoes, in domain-specific terms, much of what PPI-oriented COS development now treats as a methodological standard.

Dodd et al. (2022) analyzed published COS studies and found that those developed with PPI were significantly more likely to include life impact domains, including autonomy, well-being, and community participation, than those developed solely by researchers and clinicians. These domains are more closely aligned with quality-of-life indicators and tend to be underrepresented when outcome selection is driven primarily by measurement convenience or clinical tradition (Dodd et al., 2022). For behavior analysis, this finding carries a specific implication. A field that prioritizes direct observation and experimental control may, without deliberate attention to stakeholder priorities, tend towards outcomes that are tractable to measure rather than outcomes that matter most to the people receiving support. Engagement with COS processes offers a structural mechanism for guarding against this, not by displacing the scientific rigor of behavior-analytic methodologies, but by developing processes to align the domains being measured with what stakeholders regard as meaningful outcomes.

The alignment between COS development principles and behavior-analytic constructs of social validity does not resolve the tensions between the two frameworks. COS methods were developed largely within medical research, where diagnostic criteria and clinical outcomes are often treated as relatively stable across individuals. Behavioral phenomena, by contrast, are context dependent and functionally heterogeneous in ways that can sit uneasily with cross-study standardization. Although these tensions are real, they are not a sufficient reason for nonengagement. Rather, they point to the need for behavior analysis to engage with COS development actively and on its own methodological terms, thereby contributing the measurement expertise and design conventions that existing COS frameworks lack, and where necessary developing what might be termed behavior-analytic COS that are built from the ground up to accommodate direct measurement, functional heterogeneity, and idiographic intervention design.

## What Behavior Analysis Can Contribute to COS initiatives

Behavior analysis could bring methodological resources to COS development that are not included in existing frameworks and initiatives. The field’s emphasis on precise operational definitions, direct observation, and interobserver agreement (IOA) as indices of measurement quality represents a standard of measurement rigor that indirect, proxy-rated instruments, which currently dominate COS development, cannot replicate (Essig et al., 2023). COS frameworks have tended to rely on standardized questionnaires and clinician-rated scales. Behavior analysis offers a tradition of measurement built around the direct, repeated observation of socially significant behavior under controlled conditions with explicit criteria for evaluating measurement and for determining whether observed behavior change can be attributed to the intervention rather than to extraneous variables. SCED, with its emphasis on individual-level evidence, experimental control, and replication, adds a further methodological resource that group-designs do not provide. Rather than seeking accommodation within COS infrastructure, behavior analysis is positioned to strengthen it, by contributing measurement standards and design conventions that improve the quality and interpretability of COS-aligned evidence across disciplines.

A site of tension between behavior-analytic methodology and existing COS infrastructure is the appraisal of outcome measurement instruments. COSMIN (Consensus-based Standards for the Selection of Health Measurement Instruments) guidance is widely used during COS development to evaluate the measurement properties of candidate instruments (Mokkink et al., 2020). It is oriented primarily toward standardized instruments, questionnaires and indirect measures with established psychometric properties, and does not provide criteria for evaluating directly observed, idiographic behavioral measures or SCED-derived metrics. Shelley et al. (2024) conducted a systematic review and meta-analysis of measurement tools for behaviors that challenge and behavioral function in people with intellectual disabilities, applying COSMIN criteria throughout (Shelley et al., 2024). The review was explicitly scoped to informant-report indirect measures; direct measures were excluded from the appraisal process. The authors noted that direct measurement is widely regarded as the gold standard for measuring behavior, yet it fell outside what COSMIN criteria are designed to evaluate. The result is a synthesis of measurement quality in this critical clinical area that does not include the class of measurement central to behavior-analytic practice. An analogous concern has been raised in relation to the GRADE framework, which has been associated with the downgrading of behavior-analytic studies in autism and intellectual disability research, primarily because GRADE’s certainty rating system was developed for randomized controlled trial evidence and provides limited guidance for appraising SCED-derived findings (National Collaborating Center for Mental Health, 2015; Reichow et al., 2018; Zimmerman et al., 2018). There is a corresponding risk that COS initiatives could inadvertently reproduce this pattern.

The case for extending COSMIN guidance to accommodate direct measures and SCED-derived metrics is not solely a behavior-analytic concern. SCED, sometimes referred to as N-of-1 trials, is increasingly recognized outside of behavior analysis, in medicine, rehabilitation, and neuroscience, for its capacity to generate individual-level evidence with strong internal validity (Tanious et al., 2024). Selker et al. (2023) highlighted the expanding role of SCED in medicine as a rigorous, patient-centered methodology that can integrate direct behavioral and physiological measurement into clinical decision making, particularly in chronic and complex health conditions where group-level findings may obscure important findings at an individual-level (Selker et al., 2023). That methodology central to behavior analysis is gaining traction across disciplines strengthens rather than weakens the case for its contribution to COS instrument appraisal processes. The absence of explicit COSMIN criteria for direct observation measures is, in this context, a gap that has implications beyond behavior analysis.

Extending COSMIN guidance to accommodate direct measures would require, at minimum, that target behaviors are precisely and operationally defined, a standard that behavior analysis has applied systematically for decades (Cooper et al., 2020). Operational definitions alone are insufficient, however; measurement quality also depends on whether data are collected systematically and repeatedly over time, with sufficient sampling during both baseline and treatment phases and transparent presentation of raw data. Reliability must be demonstrated through interobserver agreement procedures, collected across phases and for a pre-specified proportion of observations, with agreement indices and acceptable thresholds reported (Essig et al., 2023). Data collection procedures should remain consistent across conditions to support valid comparison between phases, and where feasible, assessor independence or blinding should be employed to reduce observer bias (Tate et al., 2008; What Works Clearinghouse, 2020). These criteria are not novel impositions on behavior-analytic practice, because they reflect conventions already embedded in SCED reporting guidance, such as SCRIBE (Tate et al., 2016) and quality appraisal frameworks such as the What Works Clearinghouse standards. The contribution behavior analysis can make to COSMIN is, in this sense, largely a matter of making explicit what the field already does well.

## Integration Challenges: Limitations, Risks, and Mitigation strategies

A further consideration for integrating SCED evidence within COS frameworks concerns how single-case methods are applied in practice. Behavior-analytic research using SCED spans a continuum from dynamic-inductive applications, where intervention components are adjusted iteratively in response to an emerging repertoire of behavior, to static-deductive applications, where procedures and outcome criteria are specified a priori to test predefined hypotheses (Johnson & Cook, 2019; Tincani & Travers, 2019). Behavior-analytic research does not always make this distinction explicit, which may create difficulties for evidence synthesis: dynamic-inductive and static-deductive studies are evaluating different things and should be appraised on different terms, but without clear designation in reporting, reviewers have no reliable basis for distinguishing between them. Making this distinction explicit, in study reporting, in COS guidance, and in synthesis frameworks, would allow the exploratory, response-guided logic of dynamic-inductive methods to be recognized as a legitimate and distinct research strategy rather than treated as a methodological inconsistency. Reporting guidance accompanying behavior-analytic COS initiatives could specify this distinction as a minimum reporting requirement, supporting more principled interpretation of heterogeneity across the SCED literature.

Behavior analysis has also developed methods for operationalizing stakeholder involvement that extend beyond the consensus panel and Delphi survey approaches typical of COS development, an important contribution given that the population most likely to be supported by these interventions often include people with intellectual disabilities, complex communication needs, or limited verbal repertoires. Standard PPI methods may not be accessible to such individuals, which could raise questions about whose priorities are being reflected in consensus sets. Hanley (2010) addressed this directly by operationalizing social validity as a measurable construct, embedding choice and preference assessment into intervention design so that individuals, including those with limited verbal repertoires, can shape the selection and delivery of procedures based on their demonstrated preferences (Auten et al., 2024; Hanley, 2010). This approach not only accommodates stakeholder involvement; it provides a methodology for making it meaningful in populations where conventional consultation methods are insufficient. For COS development in behavior-analytic research and service context, this represents a substantive methodological contribution: a set of tools for ensuring that outcome prioritization reflects the priorities of those receiving support, rather than those most able to articulate them in a survey or consensus meeting.

Behavior-analytic COS initiatives are more likely to achieve credibility and uptake across disciplines if they align with internationally recognized standards for COS development and reporting. The Core Outcome Set Standards for Development (COS-STAD) specifies eleven minimum standards across three domains, including scope, stakeholder involvement, and consensus processes, as well as providing a practical framework that does not prescribe research design or measurement modalities, making it applicable to behavior-analytic contexts without methodological compromise (Kirkham et al., 2017). Within the scope domain, developers are expected to define the population, settings and intervention types the COS will address, a requirement that maps onto behavior-analytic practice of specifying the conditions under which findings are expected to generalize. The stakeholder involvement domain aligns with the social validity framework and the preference assessment methods discussed above, while the consensus process domain, requiring that methods be pre-specified, transparent and iterative, is consistent with behavior analysis’s emphasis on procedural transparency and data-informed decision making (Cooper et al., 2020; Wolf, 1978). The companion Core Outcome Set Standards for Reporting (Kirkham et al., 2016) supports clear and complete reporting of scope, stakeholder involvement, and consensus methods, improving transparency and reproducibility. Adopting both standards would support the dissemination of behavior-analytic COS initiatives across interdisciplinary evidence synthesis and guideline development.

A further tension between COS frameworks as presently designed and behavior analysis is not only methodological but philosophical. COS are designed to produce generalizable cross-study comparisons by standardizing what is measured across populations and settings. Behavior analysis, by contrast, takes the individual as the primary unit of analysis, a commitment that is not merely a methodological preference but central to the field’s scientific identity (Baer et al., 1968). From this perspective, an outcome that is meaningful to one individual may be irrelevant or misleading for another, even within the same diagnostic category or intervention context. The concern is not that standardization is inherently incompatible with behavior analysis, but that COS frameworks developed without sensitivity to this commitment may inadvertently privilege outcomes that are readily standardized over those that are clinically and socially meaningful at the level of the individual. Although this tension may not be fully resolvable, it may be managed by ensuring that behavior-analytic COS initiatives specify core domains at a level of abstraction that accommodates functional heterogeneity, and by treating the core domain set as the reporting floor, rather than as a constraint on what individual studies may measure.

A practical risk of engagement with COS frameworks is that standardization incentivizes reliance on indirect measures. Outcomes that are easier to standardize, including caregiver reports, clinician-scored scales, and standardized questionnaires, are more familiar within COS development infrastructure and more readily appraised using existing COSMIN criteria. Direct measures, by contrast, require additional specification and are not easily accommodated within existing instrument appraisal processes, creating a practical pressure towards indirect measurement that may be difficult to resist without deliberate structural countermeasures. A related risk is that outcomes selected for their comparability across studies may be less sensitive to the idiographic change that behavior-analytic interventions produce, particularly in the early phases of intervention, where the most clinically meaningful changes may be at the level of topography, rate, or latency rather than composite scale scores. Both risks are substantially mitigated by the domain/measurement distinction that is central to the COS framework: specifying what should be measured at the domain-level leaves the choice of measurement instrument to the researcher, preserving the option of direct observation while ensuring that shared domains are reported across studies.

**Fig. 1:**
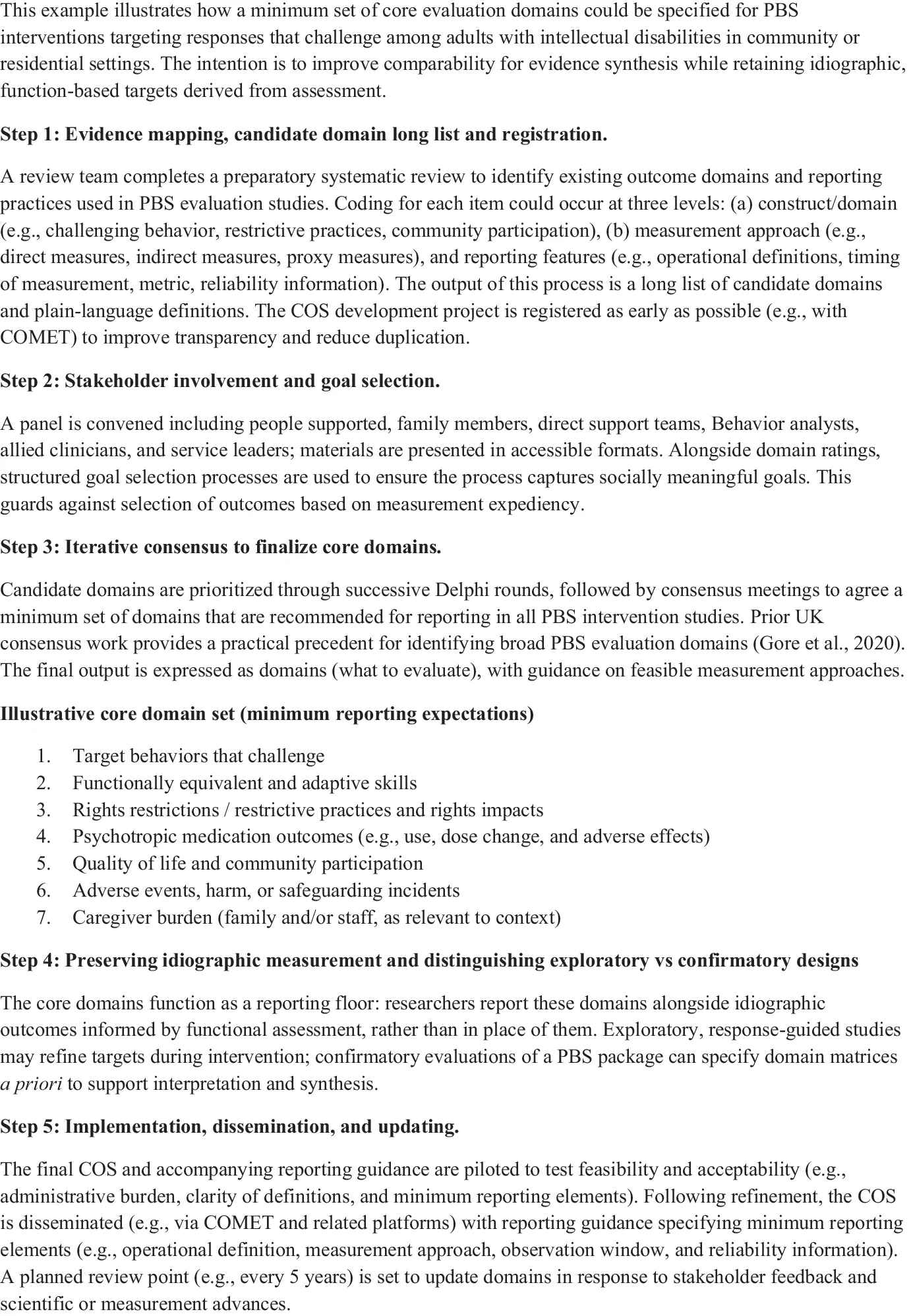
Worked example: Building a minimum set of core outcome domains for PBS studies in adult intellectual disability supports

An additional risk that directly concerns the integrity of behavior analysis is the potential marginalization of idiographic, process-oriented measures, informed by functional assessment. Functional assessment identifies the contextual variables that bear a functional relationship to operationally defined target responses, and the intervention targets it informs are person-specific in ways that cannot be captured by domain-level standardization. A COS that specifies challenging behavior as a core outcome domain, for example, does not and cannot specify the function-based intervention targets that functional assessment informs for a given individual: two people may present with topographically similar responses maintained by entirely different variables, requiring different intervention approaches and producing different outcomes. The risk is not that COS frameworks will prohibit such measures, but that reporting expectations and instrument appraisal processes may increasingly marginalize them in favor of outcomes that are more readily compared across studies. The mitigation lies in how behavior-analytic COS initiatives are designed: if accompanying guidance specifies that idiographic, function-based targets are reported alongside core domains rather than in place of them, the tension between standardization and individualization becomes a design feature rather than a contradiction. Figure 1 illustrates how these principles might be applied in practice, using PBS interventions for adults with intellectual disabilities as a worked example.

## The Future for Behavior Analysis and COS

Several concrete steps follow from the positions articulated in this article. The first concerns engagement with existing multidisciplinary COS initiatives rather than the development of behavior-analytic COS in isolation. Joseph et al. (2024) ICHOM autism COS is a concrete example of this and represents an existing framework developed with behavior-analytic input. There is both an opportunity and an obligation for the field to engage with its implementation, evaluate its adequacy for behavior-analytic service contexts, and contribute to its updating as the evidence base develops. Developing complementary behavior-analytic COS without engaging with existing initiatives risks duplication and may reduce rather than increase the field’s influence on clinical guideline and policy development.

The second step is methodological advocacy within COS infrastructure. The case for extending COSMIN guidance to accommodate direct measures and SCED-derived metrics has been made in this article but making that case in the literature is likely to be insufficient without active participation in the working groups and consensus processes through which COSMIN guidance is developed and revised. Behavior analysts with expertise in direct measurement and SCED are well positioned to contribute to the process, and the growing recognition of N-of-1 methodologies across disciplines provides a favorable context for this advocacy.

The third step is empirical. Advancing this agenda requires systematic examination of outcome measurement practices within specific intervention literatures to map current approaches, identify gaps, and generate the evidence base needed to inform stakeholder-led COS development. As an example of this approach, we are currently undertaking a review of outcome measurement in PBS intervention research for adults with intellectual disabilities who experience challenging behavior to support COS development in this area.

## Conclusion

This article has provided evidence that outcome heterogeneity may constrain the advancement of evidence synthesis in behavior analysis in ways that are not immediately visible at the level of individual studies. Variability in what is measured, how it is measured, and when assessments are conducted reduces comparability across studies, complicates meta-analytic integration, and weakens the evidential base available to inform policy and practice. This occurs not because individual studies are poorly conducted, but because the evidence-base has developed without sufficient coordination in outcome selection and reporting. COS frameworks offer a collaborative response to this challenge, and the argument developed in this article suggests that behavior analysis has both the methodological resources and the practical incentives to engage with and contribute to the COS infrastructure. COS development processes, grounded in systematic stakeholder involvement, provide a structured and replicable approach to integrating social validity into outcome selection. In doing so, they address a longstanding commitment within behavior analysis that has not been consistently operationalized across the intervention research literature. This article has argued that engagement with COS should proceed in a manner that explicitly acknowledges associated risks, and that those risks should be clearly articulated and addressed by researchers in the field of behavior analysis. A key question is whether the field can engage with COS infrastructure in ways that preserve its idiographic and function-based commitments while improving the comparability and policy relevance of its evidence base. The field’s engagement with COS frameworks raises an important question regarding whether the evidence behavior analysis has accumulated over decades continues to inform clinical guidelines and policy decisions that determine the supports people receive.

## Data Availability

No datasets were generated or analysed during the current study.

## Footnotes

1 This article uses the term *challenging behavior* because it is the terminology adopted in the NICE NG11 2015. Other terms such as *behaviors that challenge*, *concerning behavior* or *behavioral expressions of need* are also used in the literature and in practice to describe the same construct. Use of the term *challenging behavior* is not intended to privilege one framing over another, but to ensure consistency with established guideline terminology.
